# Impact of COVID-19 Infection Among Heart Transplant Recipients: A Southern Brazilian Experience

**DOI:** 10.3389/fmed.2022.814952

**Published:** 2022-02-09

**Authors:** Fernando Luis Scolari, Laura Caroline Tavares Hastenteufel, Lídia Einsfeld, Julia Bueno, Letícia Orlandin, Nadine Clausell, Lívia Adams Goldraich

**Affiliations:** ^1^Heart Transplant Program, Division of Cardiology, Hospital de Clinicas de Porto Alegre, Porto Alegre, Brazil; ^2^Ted Rogers Center for Heart Research, Peter Munk Cardiac Center, University Health Network, Toronto, ON, Canada; ^3^Division of Internal Medicine, Hospital de Clinicas de Porto Alegre, Porto Alegre, Brazil; ^4^Pharmacy Service, Clinical Pharmacy Section, Hospital de Clinicas de Porto Alegre, Porto Alegre, Brazil; ^5^Faculty of Medicine, Universidade Federal do Rio Grande do Sul, Porto Alegre, Brazil

**Keywords:** heart transplant, COVID-19, Brazil, immunosuppression, infection

## Abstract

**Purpose:**

The coronavirus-2019 (COVID-19) infection is associated with a high risk of complications and death among heart transplant recipients. However, most cohorts are from high-income countries, while data from Latin America are sparse.

**Methods:**

This is a retrospective cohort of heart transplant recipients followed at a hospital in Rio Grande do Sul, Brazil, between March 1st 2020 and October 1st 2021.

**Results:**

Of the 62 heart transplant recipients on follow-up, 21 (34%) were infected by COVID-19, 58 (36–63) years of age, 67% male, body mass index of 26 (23-29) kg/m^2^, 48% with hypertension, 43% with chronic kidney disease, 5% with diabetes, within 2 (1–4) years of post-transplant follow-up. At presentation, the main symptoms were fever (62%), myalgia (33%), cough (33%), headache (33%), and dyspnea (19%). Hospitalization was required for 13 (62%) patients, with a time from first symptoms to the admission of 5 (1–12) days. In 38%, supplementary oxygen was needed, 19% required intensive care, and 10% mechanical ventilation. Three (14%) were infected after at least a first dose of COVID-19 vaccine. The main complications were bacterial pneumonia (38%), renal replacement therapy (19%), sepsis (10%) and venous thromboembolism (10%). Immunosuppression therapy was modified in 48%, with a reduction in the majority (89%). Two (10%) patients died in the hospital due to refractory hypoxemia and multiple organ dysfunction. The incidence of COVID-19 among transplant patients was comparable to the general population in the State of Rio Grande do Sul with a peak in December 2020.

**Conclusion:**

Heart transplant recipients shown a high rate of COVID-19 infection in Southern Brazil, with typical symptom presentation in most cases. There was an elevated rate of hospitalization, supplementary oxygen support, and complications. In-hospital lethality among infected heart transplanted recipients was similar to previously reported data worldwide despite the high rates of infection in Latin America.

## Introduction

The SARS-COV-2 pandemic has deeply impacted our health care systems and caused almost 5 million deaths worldwide (1). Since its beginning in 2019, there was an urgency toward a comprehensive understanding of the risk factors related to the severe presentation of coronavirus-2019 (COVID-19) infection, resulting in a high frequency of complications and death (2). Although viral pneumonia is responsible for the central hypoxic respiratory insufficiency that may lead to acute respiratory distress syndrome, several other organs may be affected, increasing the disease burden (2, 3).

Solid organ transplant recipients are at special risk for developing severe forms of COVID-19 infection and subsequent increased mortality (3, 4). Maintenance immunosuppression regimens following heart transplant usually require the use of multiple drugs and/or higher therapeutic levels in comparison to other solid organ transplants, which increases the risk for both opportunistic and non-opportunistic infections (5). Previous data have shown that lethality secondary to COVID-19 infection among heart transplant recipients can reach up to 33% in some series (4). However, infection rates and lethality may also be associated with local incidence, transplant drug protocols, clinical characteristics, and other preexisting concomitant comorbidities (5). Latin America is an endemic region for several infectious diseases, such as Chagas, Dengue fever, malaria, and was severely impacted by the COVID-19 pandemic (6). In Brazil, is estimated that by October 2021 over 20 million individuals have been infected, representing around 10% of the population (1). In this scenario, heart transplant recipients are at high risk of being infected by the SARS-COV-2 virus. Up to now, data on the natural history of COVID-19, including clinical presentation and outcomes, among heart transplant recipients were mainly reported for European and North American populations (3–5). The present study aimed to evaluate the incidence, clinical manifestations, and outcomes of heart transplant recipients infected with COVID-19 in a transplant center in Brazil and to compare our data with the international literature.

## Methods

### Population Selection

This is a retrospective cohort study of heart transplant recipients followed at Hospital de Clínicas de Porto Alegre, a tertiary academic, public institution with a low volume heart transplant center (reported 1 year survival around 90%) in Porto Alegre, Southern Brazil (7). We retrospectively evaluated all patients with signs and symptoms compatible with COVID-19 infection confirmed by a polymerase chain reaction (PCR) exam with a positive result for the detection of SARS-COV-2 between March 2020 and October 2021. All included patients were transplanted and followed in the long term at our institution. Electronic patients' charts and clinical records were reviewed by a staff physician of the transplant team, and the following variables were collected: demographics, comorbidities, vaccination history, symptoms at the diagnosis of COVID-19 infection, hospital admissions, in-hospital treatments, complications, and death. Patients with a clinical suspicion but without a positive COVID-19 test were excluded from this analysis.

### COVID-19 Management

All COVID-19 infected heart transplant recipients that required hospitalization at our institution were followed by the heart transplant team, the transplant infectious diseases team, and respirology team. Our patients were also followed remotely by the transplant staff. Immunosuppression decisions to increase or reduce therapy were made by our heart transplant team. Adjuvant therapy to COVID-19 was used according to evidence-based treatments available locally, following our institution's protocol. Briefly, all hospitalized patients with the need for oxygen support were treated with dexamethasone 6 mg per day for up to 10 days, even in patients on chronic corticosteroid treatment.

### Regional and International Data Comparison

The incidence of COVID-19 infection among heart transplant recipients was compared to that observed overall in the Rio Grande do Sul, the Southernmost state of Brazil with an estimated population of 12 million. Statewide COVID-19 pandemic statistics were obtained from public records provided by the Rio Grande do Sul Health Department available at https://ti.saude.rs.gov.br/covid19/ and assessed on October 21st, 2021. We also performed a non-systematic review of case series and cohorts reporting incidence, clinical manifestations and outcomes of COVID-19 infection in heart transplant recipients worldwide to provide a comparative perspective with our local Brazilian data.

### Statistical Analysis

Continuous data were presented as median (p25-p75), while categorical data were shown as absolute counts and relative frequencies. The frequency of COVID-19 cases in the state of Rio Grande do Sul were plotted for the same period as heart transplant recipient's infection observed at our institution and a time trend analysis was performed. All analyses were performed with SPSS Version 25.0 for Windows (SPSS Inc., Chicago, IL, USA).

## Results

During the study period, 21 (24%) of the 62 heart transplant recipients in follow-up at our center developed clinical manifestations of COVID-19 infection with diagnostic confirmation by positive PCR testing. The median age of infected patients was 58 (36–63) years, with 10 (48%) being older than 60 years and 14 (67%), male. Body mass index was 26.0 (22.7–29.1) kg/m^2^. The main comorbidities were hypertension in 10 (48%), stage 3 chronic renal insufficiency in 9 (43%), pre-transplant smoking in 5 (24%), and diabetes in 1 (5%). The most common pre-transplant etiology was ischemic cardiomyopathy in 6 (29%). One patient had Chagas disease concomitant to ischemic cardiomyopathy, but no history of post-transplant reactivation. Maintenance immunosuppressive regimen consisted of tacrolimus in 17 (81%), everolimus/sirolimus in 11 (52%), mycophenolate in 10 (48%), and prednisone in 16 (76%). The time from the heart transplant to COVID-19 infection was 2 (1–4) years. In 3 (14%) patients, a rejection episode was treated within 3 months before the development of COVID-19. In our region, vaccination among solid organ transplant patients was initiated in April 2021. Table 1 summarizes the clinical characteristics of the study cohort.

**Table 1:** Demographics and clinical characteristics at symptoms presentation of heart transplant recipients diagnosed with COVID-19 infection.

Age, years	58 (36–63)
Age > 60 years	10 (48%)
Male sex	14 (67%
BMI, kg/m^2^	26 (23–29)
Hypertension	10 (48%)
Diabetes	1 (5%)
CKD	9 (43%)
COPD	1 (5%)
Previous smoker	5 (24%)
Pre-transplant ischemic cardiomyopathy	6 (29%)
**Immunosuppression therapy**	
→ Tacrolimus	17 (81%)
→ Cyclosporine	3 (14%)
→ Mycophenolate	10 (48%)
→ Azathioprine	1 (5%)
→ Everolimus/sirolimus	11 (52%)
→ Time from transplant, years	2 (1–4)
**COVID-19 clinical presentation**	
Fever	13 (62%)
Headache	7 (33%)
Myalgia	7 (33%)
Cough	7 (33%)
Rhinorrhea	4 (19%)
Odynophagia	3 (14%)
Diarrhea	5 (24%)
Anosmia	2 (10%)
Dyspnea	4 (19%)
Hemoglobine, g/dL	10.7 (8.4–13.1)
Hematocrit, %	31 (26–40)
Leukocyte, μL, ×10^3^	4.1 (2.1–6.4)
Creatinine, mg/dL	1.7 (1.2–2.9)
LDH, units/L	389 (235–562)
C-reactive protein, mg/L	73 (49–224)
D-dimer, μg/mL	0.89 (0.37–2.98)

*BMI, body mass index; CKD, chronic kidney disease; COPD, chronic obstructive pulmonary disease; LDH, lactate dehydrogenase*.

### COVID-19 Manifestations and Management

The most frequent symptoms presented by heart transplant recipients in our cohort were fever in 13 (62%), headache in 7 (33%), myalgia in 7 (33%) and cough in 7 (33%). Other less frequently observed complaints were diarrhea in 5 (24%), rhinorrhea in 4 (19%), odynophagia in 3 (14%), and anosmia in 2 (10%). Dyspnea occurred in only 4 (19%) at the initial presentation. The time from symptom onset to COVID-19 infection diagnosis was 4 (1–7) days. There were no cases of asymptomatic COVID-19 detected during the study period. Table 1 summarizes the laboratory evaluation of heart transplant recipients at clinical presentation. There were no significant differences in test values between patients admitted to the hospital vs. those who were managed as outpatients (*data not shown*). The immunosuppression regimen was modified in 10 (48%) patients, all of whom were hospitalized. Of those, nine had a reduction in the number and/or doses of immunosuppressive agents. Mycophenolate was the most frequently reduced or discontinued agent (6 patients), followed by tacrolimus in one. There were no cases of immunosuppression withdrawal. No patients developed acute graft rejection during the symptomatic period or in the subsequent 3 months. Corticosteroid therapy for both patients hospitalized and managed remotely followed institutional protocols for COVID-19, and challenging cases were eventually reviewed by the transplant infectious disease team for prescription of dexamethasone course. No patients received other specific therapies for COVID-19, such as remdesivir or tocilizumab, as they are not yet widely available for use in the public health care system in Brazil.

Three (14%) heart transplant recipients developed COVID-19 post-vaccine. One patient had only one dose and two had two doses before being diagnosed with active infection. In one case, the disease manifested 5 days after the second dose. All three patients had received NT162b2 CoronaVac (Sinovac Life Sciences, Beijing, China) vaccine (9). Table 2 summarizes the clinical presentation and disease course of the post-vaccination COVID-19 cases. Briefly, they presented typical symptoms that varied among patients. All required hospitalization, but only one needed oxygen support and developed secondary pneumonia. All three patients survived. By the end of the study period, all heart transplant recipients were fully vaccinated for COVID-19.

**Table 2:** Clinical characteristics and disease course of heart transplant recipients with COVID-19 infection following vaccination.

**#**	**Age (years)**	**Sex**	**Number of vaccines of doses before COVID-19 infection**	**Time from the last vaccine shot to COVID-19 diagnosis (days)**	**Comorbidities**	**IS therapy**	**Symptoms at presentation**	**Disease course**	**Alive**
1	47	M	1	46	HTN, CKD, dyslipidemia	TAC, EVL, steroid	Fever, odynophagia, rhinorrhea, headache	Hospitalized in ICU, acute non-biliary pancreatitis with diabetic ketoacidosis, non-oliguric acute kidney injury	Yes
2	70	M	2	5	HTN, CKD	CsA, EVL, steroid	Fever, cough, dyspnea	Hospitalized in ICU, need for the high-flow nasal cannula, complicated by bacterial pneumonia	Yes
3	63	F	2	36	HTN, mitral valve regurgitation	TAC, MMF, steroid	Fever, myalgia, diarrhea	Hospitalized inward, no oxygen therapy requirement	Yes

*IS, immunosuppression; M, male; F, female; CKD, chronic kidney disease; HTN, hypertension; EVL, everolimus; MMF, mycophenolate; TAC, tacrolimus, CsA, cyclosporine; ICU, intensive care unit*.

### In-hospital Follow-Up and Outcomes

Of 21 heart transplant recipients who were infected by COVID-19, 13 (62%) required hospitalization during their symptomatic course. The time from symptom onset to hospital admission was 5 (1–12) days. Only one patient presented to the hospital with a peripheral oxygen saturation lower than 92%. During the hospitalization, 8 (62%) required supplementary oxygen therapy: nasal cannula was used in 7 (88%); Hudson mask, in 6 (75%); high flow nasal cannula, in 4 (50%); bi-level positive airway pressure (BiPAP), in 1 (13%); and invasive mechanical ventilation, in 2 (25%).

In-hospital complications occurred in 10 (77%) patients. The most common complication was concomitant bacterial pneumonia in 8 (62%), followed by renal replacement therapy in 4 (31%), sepsis in 2 (16%), opportunistic infection in 2 (16%), deep venous thrombosis in 1 (8%), and pulmonary embolism in 1 (8%). During their hospital stay, 4 (31%) patients required transfer to intensive care units. For all of them, the reason for escalating care intensity was hypoxia and the need for greater respiratory support. All 4 patients required renal replacement therapy. Two patients were successfully discharged home, but two died while in the intensive care unit due to refractory hypoxemia and multiple organ dysfunction. In addition to the heart transplanted status, patients who died had significant preexisting comorbidities—one had moderate chronic obstructive pulmonary disease and the other had chronic kidney disease treated with hemodialysis for a year. The length of hospitalization was 17 (8-28) days. Survival was 90% for the overall cohort, and 85% for hospitalized patients.

### Comparison to the Overall Regional Population

**Figure 1:**
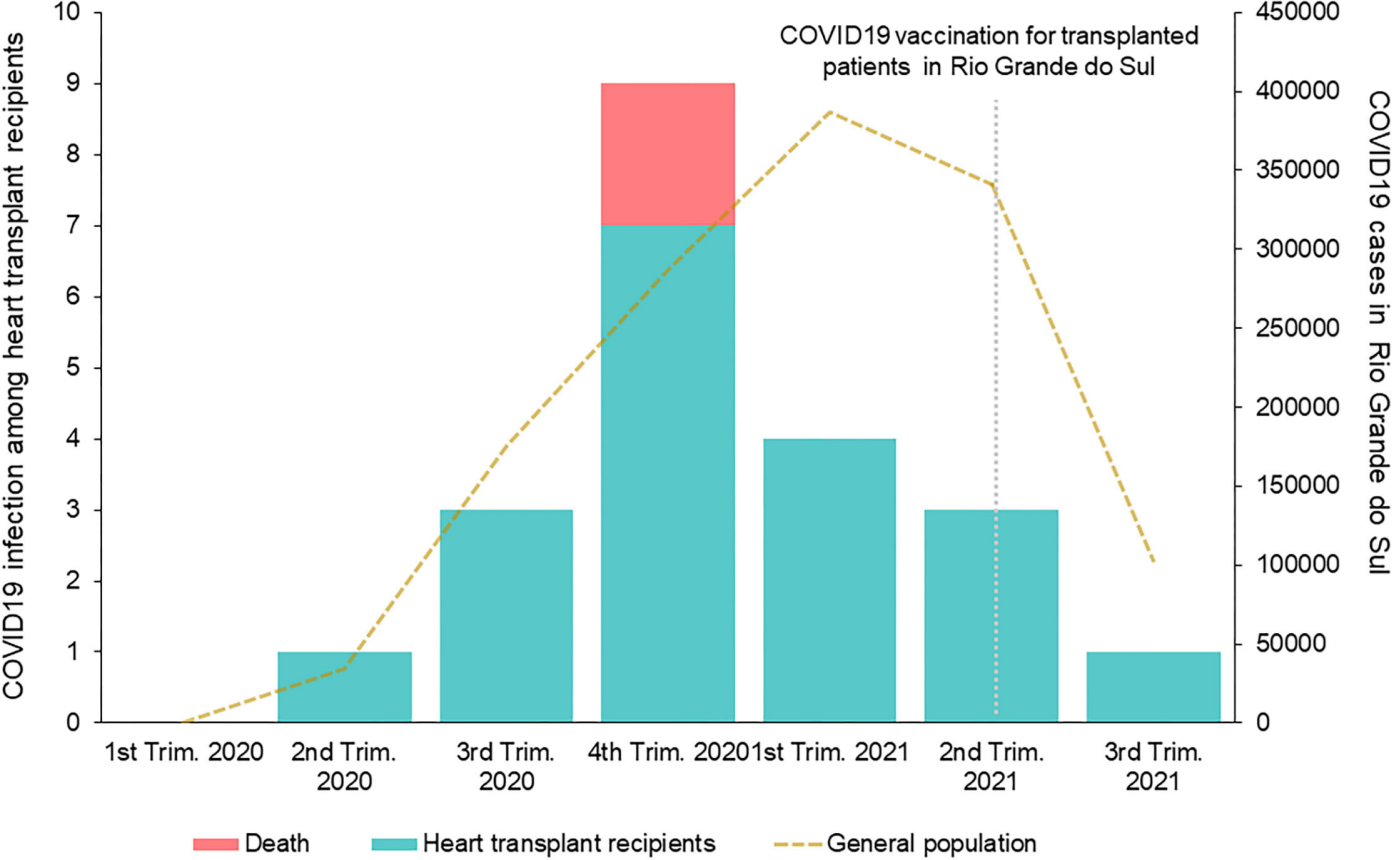
Temporal incidence of COVID-19 infection in the general population and the heart transplant recipient cohort in the State of Rio Grande do Sul.

Figure 1 depicts the overall incidence of COVID-19 infection in the state of Rio Grande do Sul and in the study cohort of heart transplant recipients. In the heart transplant recipients, the peak incidence was in the 4th trimester of 2020, while there was a high incidence in the general population that peaked in the first trimester of 2021.

### Comparison With International Cohorts of Heart Transplant Recipients

Table 3 summarizes clinical characteristics and outcomes of heart transplant recipients infected with COVID-19. Previously published cohorts of heart transplant recipients included a range of 2 to 99 (total of 341) patients evaluated from January 2020 to February 2021. There was a wide range of symptoms at presentation whereas fever was observed in 25–100% of cases and dyspnea, in 20–100%. In nearly all cohorts, some degree of reduction in immunosuppression during the infectious course was described. Only one paper reported graft rejection in the context of immunosuppression withdrawal. Although some articles only included hospitalized patients, the lowest hospital admission frequency among COVID-19 infected heart transplant recipients was 50%. Lethality rates ranged from none up to 33%.

**Table 3:** Demographics, clinical characteristics and outcomes from previously published cohorts of heart transplant recipients infected with COVID-19.

**References**	**Country**	**N**	**Date**	**Time from TX**	**Dyspnea**	**Cough**	**Myalgia**	**Fever**	**IS**	**Graft rejection**	**Hosp**.	**Invasive ventilation**	**Death**
Li et al. (11)	China	2	Jan–Feb 2020	3–17 years	50%	50%	50%	100%	Reduced/ withdrawal	None	100%	0%	0%
Lima et al. (8)	USA	5	Mar–Apr 2020	13–604 days	60%	100%	NA	60%	Reduced	None	100%	40%	None
Rivinus et al. (4)	Germany	21	Mar–Jun 2020	2,858 ± 2,516 days	86%	76%	76%	67%	Reduced	None	91%	38%	33%
Al-Darzi et al. (14)	USA	6	Mar–May 2020	6.5 (4.25–12) years	100%	80%	40%	100%	Reduced	None	83%	0%	0
Singhvi et al. (21)	USA	22	Mar–May 2020	4.6 (2.5–20.6) years	NA	46%	NA	68%	Reduced	0%	86%	18%	23%
Felldin et al. (15)	Sweden	6	Feb 2020–Jun 2020	120–228 months	NA	NA	NA	NA	Reduced/ maintained	None	50%	NA	17%
Soriano et al. (20)	Brazil	11	Mar 2020–Jul 2020	3–264 months	45%	72%	NA	72%	Reduced/ withdrawal	None	100%	20%	27%
Genuardi et al. (22)	USA	99	Mar 2020–Oct 2020	5.6 (2.0–13.7) years	41%	49%	NA	51%	NA	None	64%	20%	15%
Latif et al. (13)	USA	28	Apr 2020	8.6 (4.2–14.5) years	91%	91%	NA	83%	Reduced/ withdrawal	None	79%	25%	25%
Marcondes-Braga et al. (10)	Brazil	40	Apr 2020–Jan 2021	NA	40%	NA	NA	45%	Reduced/ withdrawal	10%	82%	23%	12%
Duran et al. (9)	USA	28	Feb 2020–Feb 2021	NA	21%	36%	7%	25%	Reduced	None	54%	11%	7%
Lopez-Villela et al. (23)	Spain	20	Feb 2020–Feb 2021	1,901–3,777 days	20%	65%	NA	65%	Reduced	none	70%	10%	10%
Caraffa et al. (12)	Italy	6	NA	NA	100%	100%	83%	NA	Reduced	None	83%	33%	33%
Bottio et al. (24)	Italy	47	NA	10.46 ± 8.70 years	70%	70%	45%	87%	Reduced	None	81%	4%	30%
Our data	Brazil	21	Mar 2020–Oct 2021	2 (1–4) years	19%	33%	33%	62%	Reduced	None	62%	10%	10%

*N, number of COVID-19 infected patients; TX, transplant; IS, immunosuppression; NA, not available; Hosp., hospitalization*.

## Discussion

Heart transplant recipients are at an elevated risk for developing severe forms of COVID-19 (3, 4, 8). We identified a high incidence (24%) of the disease among our cohort of heart transplant recipients in the south of Brazil, with a typical disease presentation. Most patients required hospitalization and developed a large incidence of complications, with concomitant bacterial pneumonia frequently observed. Overall lethality among infected patients was 10%.

Subclinical manifestations of COVID-19 among heart transplant recipients leading to a late diagnosis of infection and, subsequently, delayed health care assessment was a major concern at the beginning of the pandemic period (4). In our cohort, the median time from symptom onset to diagnosis was 4 days with most patients presenting with typical clinical manifestations, such as fever, headache, myalgia, and cough. However, non-transplanted patients appear to have higher rates of typical symptoms, reaching >90% of fever and 80% of cough, especially among those that are hospitalized (2, 5, 6). As detailed in Table 3, previously published cohorts reported a wide range of symptoms' frequency among heart transplant recipients infected with COVID-19, in whom fever ranged from 25 to 100% (8–11), and cough from 36 to 100% (9, 10, 12–14). Interestingly, we did not observe dyspnea to be a major complaint at the diagnosis, which was different from other studies. The wide variability among the reported symptom proportions might reflect different inclusion criteria and study designs. In our cohort, no asymptomatic patients were included since we did not routinely screen patients for COVID-19 at our institution. We might speculate that, due to the lower rate of clinical manifestations in our cohort, a larger number of heart transplant recipients could have been infected but not diagnosed.

Immunosuppression of heart transplant recipients favors severe forms of infection, but its reduction could lead to rejection episodes, a risk potentially exacerbated by the increase of cytokine release during COVID-19 infection (5, 10, 13, 15). In our cohort, most patients had their immunosuppression therapy reduced and no rejection episodes were detected during the period. Cardiac allograft rejection during symptomatic COVID-19 or in the post-infection period was reported only in one study, in which immunosuppression was decreased or withdrawn (10). The current strategy of reducing treatment seems to be safe, although there are no clinical trials evaluating immunosuppression approaches in this population to date, and it is yet unknown if such a strategy improves COVID-19 treatment and outcomes (4).

The high proportion of heart transplant recipients infected with COVID-19 requiring hospitalization in our cohort highlights the increased risk for complications in this patient population. There was a wide range of time from initial presentation to hospital admission [median of 5 (1–12) days]. This finding may be related to the small sample but may also suggest that some patients have a fast disease progression, while others have a slower course. Nearly all hospitalized patients had an in-hospital complication, and bacterial pneumonia was the most common. Concomitant bacterial infection may occur due to the structural and functional changes in the lung parenchyma caused by COVID-19. However, among non-transplant individuals with COVID-19, a lower incidence of concomitant bacterial pneumonia was described (16). The immunosuppression state could explain these findings as well as the reported elevated rate of sepsis and opportunistic infections. The spectrum of other observed complications, such as the need for renal replacement therapy, deep venous thrombosis, and pulmonary embolism, confirms the systemic involvement of moderate to severe forms of the disease.

We observed that three patients developed COVID-19 following some degree of vaccination. Those infection episodes were associated with a large burden of symptoms, need for hospitalization and complications. The small number of cases limits the interpretation of our observations. However, it has been discussed that immune paresis might be more prevalent in heart transplant recipients than in other solid organ recipients and that they may benefit from booster shots of COVID-19 vaccines to induce long-lasting protection (17). A third dose was not associated with severe reactions and promoted a positive antibody response in 67% of heart transplant recipients after 18 days. Based on those results, a third booster shot has been currently recommended by the largest transplantation societies, internationally, and has already been incorporated into vaccination policies by many countries, including Brazil (17, 18). It is also possible that the lower efficacy of the CoroVac vaccine in comparison to other vaccines might have been the major factor for the observed post-vaccination infection in our patient population (19).

A 10% lethality rate among heart transplant recipients with symptomatic COVID-19 was observed. Previous reports described death rates ranging from 0 to 33%. Early reports showed the highest lethality rates (4, 12, 20). It is unknown if the outcome differences observed over time could be related to improvements in COVID-19 treatment, such as with the use of dexamethasone, or virus variants. Previous centers' experiences in Brazil showed a lethality rate of 12.5–27% among transplant recipients, however, the study with the highest rate included only hospitalized patients (10, 20). Regional differences and local incidence may explain these variations. As shown in Figure 1, deaths were observed near the pandemic peak in our center. The elevated number of infected individuals in the overall population and the impact on the health care systems could have contributed to the adverse outcomes. As for internationally reported data involving heart transplant patients with COVID-19 (4, 8–15, 20–24), our data compared somewhat favorably with regards to lethality rates, being within reported ranges, despite very high COVID-19 infection rates in Brazil and Latin America (6).

Our study limitations include its retrospective nature and its relatively limited sample size. Also, we only considered COVID-19 infected patients those who tested positive—thus we may have missed patients who were infected but did not test. Nonetheless, we sought to share our experience in a scarcely reported field, particularly in Latin American transplant centers and compare it to larger and international studies.

## Conclusions

The incidence of COVID-19 infection among heart transplant recipients in Southern Brazil was elevated, presenting with typical clinical manifestations in most cases, and high rates of hospitalization, the requirement of supplementary oxygen, and complications. Although COVID-19 incidence in Latin America was high, in-hospital lethality among infected heart transplanted patients in our region was within the range of fatal outcomes previously reported worldwide.

## Data Availability Statement

The raw data supporting the conclusions of this article will be made available by the authors, without undue reservation.

## Ethics Statement

The studies involving human participants were reviewed and approved by Grupo de Pesquisa e Pós-graduação do Hospital de Clinicas de Porto Alegre. The patients/participants provided their written informed consent to participate in this study.

## Author Contributions

All authors listed have made a substantial, direct, and intellectual contribution to the work and approved it for publication.

## Funding

This work was supported by Fundo de Incentivo á Pesquisa (FIPE)of the Hospital de Clínicas de Porto Alegre (FIPE/HCPA). The funders had no role in study design, data collection and analysis, decision to publish or preparation of the manuscript.

## Conflict of Interest

The authors declare that the research was conducted in the absence of any commercial or financial relationships that could be construed as a potential conflict of interest.

## Publisher's Note

All claims expressed in this article are solely those of the authors and do not necessarily represent those of their affiliated organizations, or those of the publisher, the editors and the reviewers. Any product that may be evaluated in this article, or claim that may be made by its manufacturer, is not guaranteed or endorsed by the publisher.
